# Transcriptome-wide N^6^-methyladenosine methylation profile of atherosclerosis in mice

**DOI:** 10.1186/s12864-023-09878-1

**Published:** 2023-12-14

**Authors:** Xinbin Zheng, Bo Zhou, Yuzhen Li, Hengren Zhong, Zhengxin Huang, Minhua Gu

**Affiliations:** 1 Clinical Research Center, Hainan Provincial Hospital of Traditional Chinese Medicine, 570203 Haikou, Hainan P. R. China; 2 Department of Cardiology, Hainan Provincial Hospital of Traditional Chinese Medicine, 570203 Haikou, Hainan P. R. China; 3 Hainan Clinical Research Center for Preventive Treatment of Diseases, 570203 Haikou, Hainan P. R. China; 4 Hospital of Chinese Medicine affiliated by Hainan Medical University, 570203 Haikou, Hainan P. R. China

**Keywords:** Atherosclerosis, N^6^-methyladenosine, MeRIP-seq, Fabp5

## Abstract

**Background:**

Atherosclerosis (AS) is a critical pathological event during the progression of cardiovascular diseases. It exhibits fibrofatty lesions on the arterial wall and lacks effective treatment. N^6^-methyladenosine (m^6^A) is the most common modification of eukaryotic RNA and plays an important role in regulating the development and progression of cardiovascular diseases. However, the role of m^6^A modification in AS remains largely unknown. Therefore, in this study, we explored the transcriptome distribution of m^6^A modification in AS and its potential mechanism.

**Methods:**

Methylation Quantification Kit was used to detect the global m^6^A levels in the aorta of AS mice. Western blot was used to analyze the protein level of methyltransferases. Methylated RNA immunoprecipitation with next-generation sequencing (MeRIP-seq) and RNA sequencing (RNA-seq) were used to obtain the first transcriptome range analysis of the m^6^A methylene map in the aorta of AS mice, followed by bioinformatics analysis. qRT-PCR and MeRIP-qRT-PCR were used to measure the mRNA and m^6^A levels in target genes.

**Results:**

The global m^6^A and protein levels of methyltransferase METTL3 were significantly increased in the aorta of AS mice. However, the protein level of demethylase ALKBH5 was significantly decreased. Through MeRIP-seq, we obtained m^6^A methylation maps in AS and control mice. In total, 26,918 m^6^A peaks associated with 13,744 genes were detected in AS group, whereas 26,157 m^6^A peaks associated with 13,283 genes were detected in the control group. Peaks mainly appeared in the coding sequence (CDS) regions close to the stop codon with the RRACH motif. Moreover, functional enrichment analysis demonstrated that m^6^A-containing genes were significantly enriched in AS-relevant pathways. Interestingly, a negative correlation between m^6^A methylation abundance and gene expression level was found through the integrated analysis of MeRIP-seq and RNA-seq data. Among the m^6^A-modified genes, a hypo-methylated but up-regulated (hypo-up) gene Fabp5 may be a potential biomarker of AS.

**Conclusions:**

Our study provides transcriptome-wide m^6^A methylation for the first time to determine the association between m^6^A modification and AS progression. Our study lays a foundation for further exploring the pathogenesis of AS and provides a new direction for the treatment of AS.

**Supplementary Information:**

The online version contains supplementary material available at 10.1186/s12864-023-09878-1.

## Background

Atherosclerosis (AS) is the major underlying cause of cardiovascular diseases such as coronary heart disease, carotid artery disease, and peripheral arterial disease [1]. AS is now considered an arterial disease characterized by the progressive accumulation of lipids, smooth muscle cells, inflammatory cells, and connective tissue in the muscular arterial intima to form lipid plaques [2, 3]. Although we have made further progress in understanding the molecular mechanisms of AS and the treatment of cardiovascular disease, the incidence of cardiovascular disease remains high. Therefore, a more in-depth and comprehensive explanation of the pathogenesis of AS will help to find more effective treatment drugs.

Increasing evidence suggests that epigenetic modifications at histone, DNA, and RNA play important roles in the pathogenesis of atherosclerosis [4]. Among them, RNA modification, a common post-transcriptional modification, has attracted increasing attention as a novel gene regulatory mechanism recently [5]. RNA methylation accounts for more than 60% of all RNA modifications, among which N^6^-methyladenosine (m^6^A) is the most common modification on eukaryotic RNA [6]. The m^6^A modification mainly occurs on adenine in the RRACH sequence motif and at the beginning of the 3’-UTR near the translation termination codon [7, 8]. The modification of m^6^A is executed by the core m^6^A methylase (writer) complex containing METTL3, METTL14, and WTAP, while ALKBH5 and FTO act as demethylases (eraser) to eliminate the m^6^A modification [9, 10]. In addition, m^6^A is recognized by the m^6^A binding protein (reader), which has been found to include the YTH domain protein family (YTHDF1/2/3 and YTHDC1/2) and the IGF2B family (IGF2BP1/2/3) [11, 12].

Recent studies have shown that RNA m^6^A modification has a wide range of biological functions, such as regulating RNA translocation, splicing, translation, degradation, and stability [13]. An increasing number of studies have indicated that m^6^A modification is involved in regulating the development and progression of AS. For instance, m^6^A methylase, demethylase, and reader were abnormally expressed in human carotid atherosclerotic plaques [1]. Decreased expression of METTL14 can inhibit endothelial inflammation and atherosclerosis development [14]. Furthermore, knockout of METTL14 significantly reduced the inflammatory response of macrophages and the development of atherosclerotic plaques [15]. However, the distribution and profile of m^6^A modification in the transcriptome in AS remain unknown.

In the present study, we first detected total m^6^A status in the aorta of ApoE^−/−^ mice, which were fed with a high-fat diet (HFD) and controls. Then we carried out the MeRIP-seq and RNA-seq to analyze transcriptome-wide m^6^A methylation in ApoE^−/−^-HFD and ApoE^−/−^-Ctrl mice. Finally, we performed the MeRIP-qPCR analysis to verify the data of MeRIP-seq.

## Results

### The overall m^6^A level was increased in AS mice

**Fig. 1:**
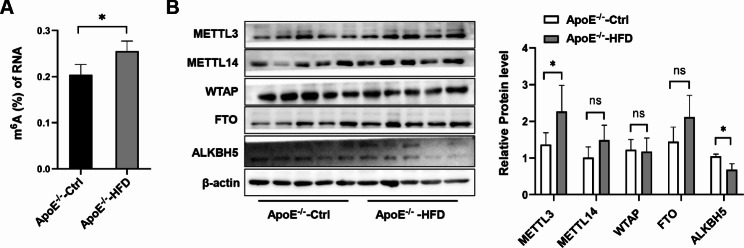
M^6^A levels in AS and control group. **(A)** The m^6^A levels in the aorta of AS and control group (n = 5). **(B)** Protein levels of the core m^6^A methylase and demethylases in the aorta of AS and control group were determined by Western blot (n = 5). The original western blot images were uploaded in Figure-1B Original Source Data and these images clearly show the membrane edges. All the data are presented as mean ± SD. *P < 0.05

To evaluate the overall m^6^A modification in atherosclerosis (AS), we first analyzed the total m^6^A level in the aorta of ApoE^−/−^ mice that were fed with high-fat diet (HFD) for 26 weeks. As shown in Fig. 1A, the total m^6^A levels in HFD-fed mice were significantly elevated when compared to the ApoE^−/−^ control mice. Due to the divergent m^6^A levels in HFD-fed and control ApoE^−/−^ mice, we further detected the expression levels of main m^6^A writers (METTL3, METTL14, and WTAP) and erasers (FTO and ALKBH5) in aorta between the two groups. Our results showed that METTL3 expression was significantly increased, ALKBH5 was significantly decreased in HFD-fed than in control mice (Fig. 1B), while the rest writers and erasers were not obviously changed. These results suggest that m^6^A modification is enhanced in AS which may be contributed by the altered expression of RNA methyltransferase and demethylase.

### Overview of m^6^A methylation modification in AS and control mice

**Fig. 2:**
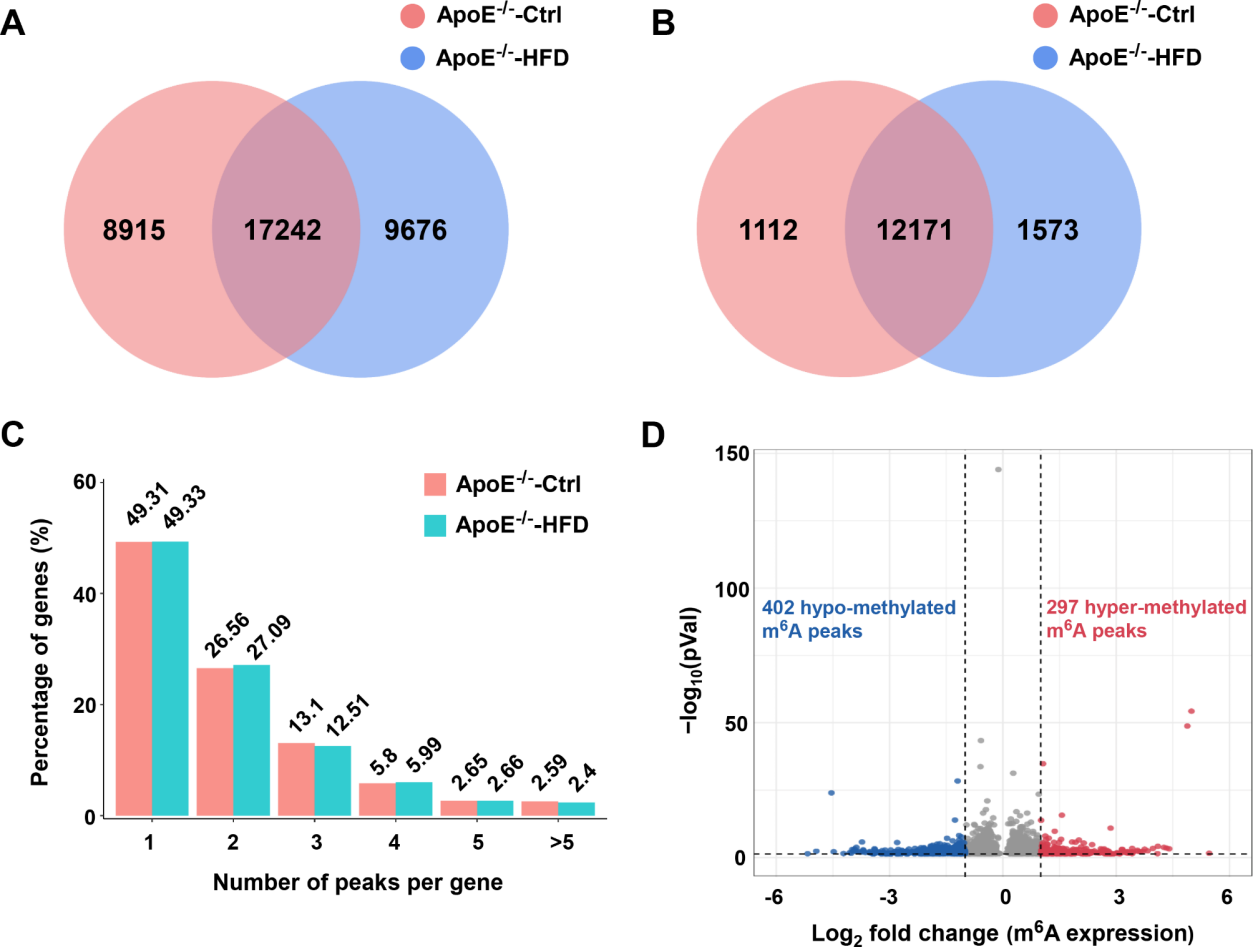
Overview of m^6^A methylation modification in AS and control group. Venn diagram showing the overlap of m^6^A peaks **(A)** and m^6^A-modified genes **(B)** between AS and control groups, respectively. **(C)** Proportion of genes harboring different number of m^6^A peaks in AS and control group. **(D)** Identification of abundance of 297 hyper-methylated and 402 hypo-methylated m^6^A peaks that showed a significant alteration (|log2 FC | > 1, *P* < 0.05), respectively, in AS group relative to that of control

To further map the profile of RNA m^6^A methylation, MeRIP-seq analysis of the aorta in AS and control mice was then performed. Data showed that 8915 nonoverlapping m^6^A peaks were related to 1112 genes in control group, 9676 nonoverlapping m^6^A peaks were related to 1573 genes in AS group, and 17,242 overlapping m^6^A peaks were related to 12,171 genes between the two groups (Fig. 2A and B). The top 20 differentially methylated m^6^A peaks were shown in Table 1. Taken together, these results indicate significant differences in m^6^A modification patterns between AS and control aorta.

**Table 1:** The top 20 differently methylated m^6^A peaks

Gene Name	Gene ID	Log_2_ Fold Change	Regulation	Chromosome	Peak start	Peak end	p-value
Gm48732	ENSMUSG00000114568	4.26	up	chr13	67,144,180	67,144,738	0
Gm15326	ENSMUSG00000086111	4.1	up	chr13	111,870,869	111,871,636	0
Gm23698	ENSMUSG00000089461	4.09	up	chr10	110,786,784	110,786,905	0.04
Gm32856	ENSMUSG00000110605	3.57	up	chr8	129,282,543	129,282,844	0
Gm16794	ENSMUSG00000097777	3.36	up	chr9	95,954,367	95,954,667	0.03
2810410L24Rik	ENSMUSG00000075389	3.32	up	chr11	120,187,527	120,187,648	0.01
Zfp85os	ENSMUSG00000044081	3.31	up	chr13	67,754,601	67,754,870	0.01
Gm28221	ENSMUSG00000101671	3.3	up	chr15	37,360,907	37,361,117	0.02
Gm6533	ENSMUSG00000115193	3.29	up	chr15	23,473,998	23,474,418	0.01
Rps15a-ps4	ENSMUSG00000083757	3.16	up	chr4	132,221,084	132,221,415	0
Gja5	ENSMUSG00000057123	-5.17	down	chr3	97,075,829	97,075,980	0.04
Mid1	ENSMUSG00000035299	-4.94	down	X	169,989,159	169,989,730	0
Rtl8b	ENSMUSG00000067924	-4.54	down	X	53,670,202	53,670,408	0
Mid1	ENSMUSG00000035299	-4.48	down	X	169,989,849	169,990,600	0.01
Gm16270	ENSMUSG00000089775	-4.22	down	chr10	86,910,167	86,910,768	0.03
Ppm1d	ENSMUSG00000020525	-4.05	down	chr11	85,311,363	85,311,543	0.01
Neu4	ENSMUSG00000034000	-4.02	down	chr1	94,009,335	94,009,546	0.03
Tspoap1	ENSMUSG00000034156	-3.99	down	chr11	87,761,169	87,761,440	0
Slc19a3	ENSMUSG00000038496	-3.95	down	chr1	83,013,414	83,013,624	0
Gm20039	ENSMUSG00000109923	-3.93	down	chr18	90,591,044	90,591,401	0

To characterize the number of m^6^A peaks on each transcript, we analyzed the results of MeRIP-seq and observed that nearly 50% of m^6^A methylated transcripts had one m^6^A peak in the two groups, and a large number of genes (89%) had 1 to 3 m^6^A-modified peaks (Fig. 2C; Table S2). Next, we analyzed the abundance of m^6^A peak between AS and control aorta. A total of 297 hyper-methylated m^6^A peaks and 402 hypo-methylated m^6^A peaks were identified in the AS aorta relative to that of control according to the criteria of |log_2_ FC| > 1 and *P* < 0.05 (Fig. 2D; Table S3).

### Distribution and topology pattern of m^6^A peaks within transcripts

**Fig. 3:**
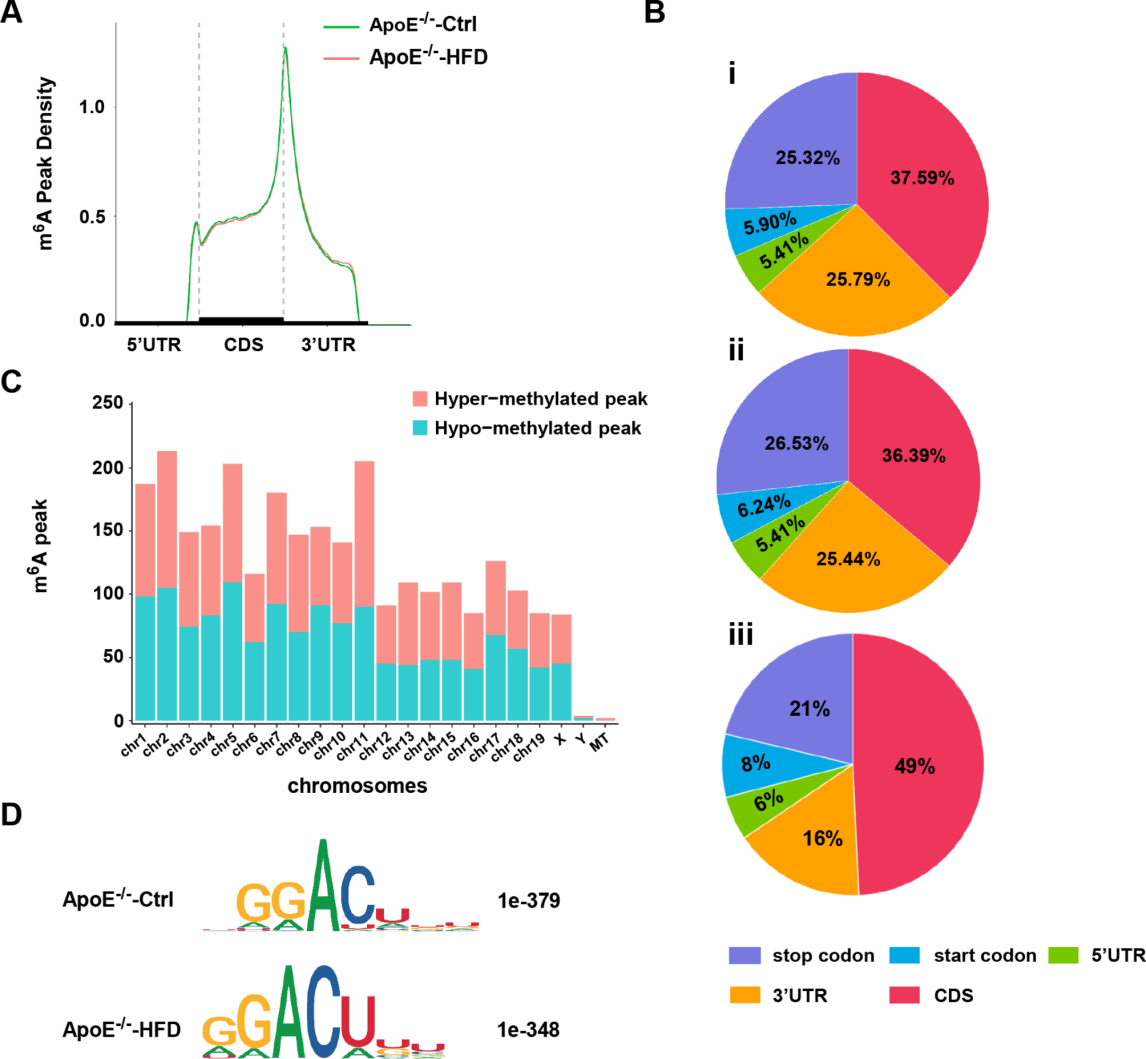
Distribution and topology pattern of m^6^A peaks along transcripts and chromatin in AS and control group. **(A)** The enrichment of m^6^A peaks along transcripts in two groups. Each transcript was divided into three parts: 5’-UTR, CDS, and 3’-UTR. **(B)** Pie charts showing the percentage of m^6^A peaks in AS group (i), m^6^A peaks in control group (ii), and differentially methylated m^6^A peaks between AS and control group (iii). **(C)** Distribution of differentially methylated m^6^A peaks with significance along the chromosome of AS mouse. **(D)** Identification of the most abundant motifs from m^6^A peaks in AS and control group, containing RRACH sequences

We next analyzed the distribution patterns of m^6^A peak along the whole transcript. The results showed that the identified m^6^A peaks mainly appeared in the coding sequence (CDS) regions close to the stop codon in AS and control groups (Fig. 3A). We then divided the transcript into the start codon, stop codon, 5’-UTR, 3’-UTR, and coding DNA sequence (CDS) to specify the location of peaks more specifically. The results showed that m^6^A peaks were significantly enriched in the CDS, 3′-UTR, and stop codon in both groups (Fig. 3B i, Fig. 3B ii), which was consistent with a previous study [16, 17]. Besides, the differentially methylated m^6^A peaks between the two groups mainly appeared in the CDS (49%), followed by the stop codon (21%) and 3′-UTR (16%) (Fig. 3B iii). The differentially methylated m^6^A peaks were further mapped to all mice chromosomes to observe their chromosomal distribution profiles. As shown in Fig. 3C, the top three chromosomes with the largest difference in methylated m^6^A peaks within transcripts were chr2, chr5, and chr11. In addition, m^6^A motif analysis showed that the classic RRACH motif existed in the region of m^6^A peaks in both groups (Fig. 3D). Collectively, the above results confirmed the reliability of our data and displayed the distribution patterns of m^6^A modification in transcripts of aorta from AS and control mice.

### Bioinformatic analysis of differentially methylated m^6^A genes

**Fig. 4:**
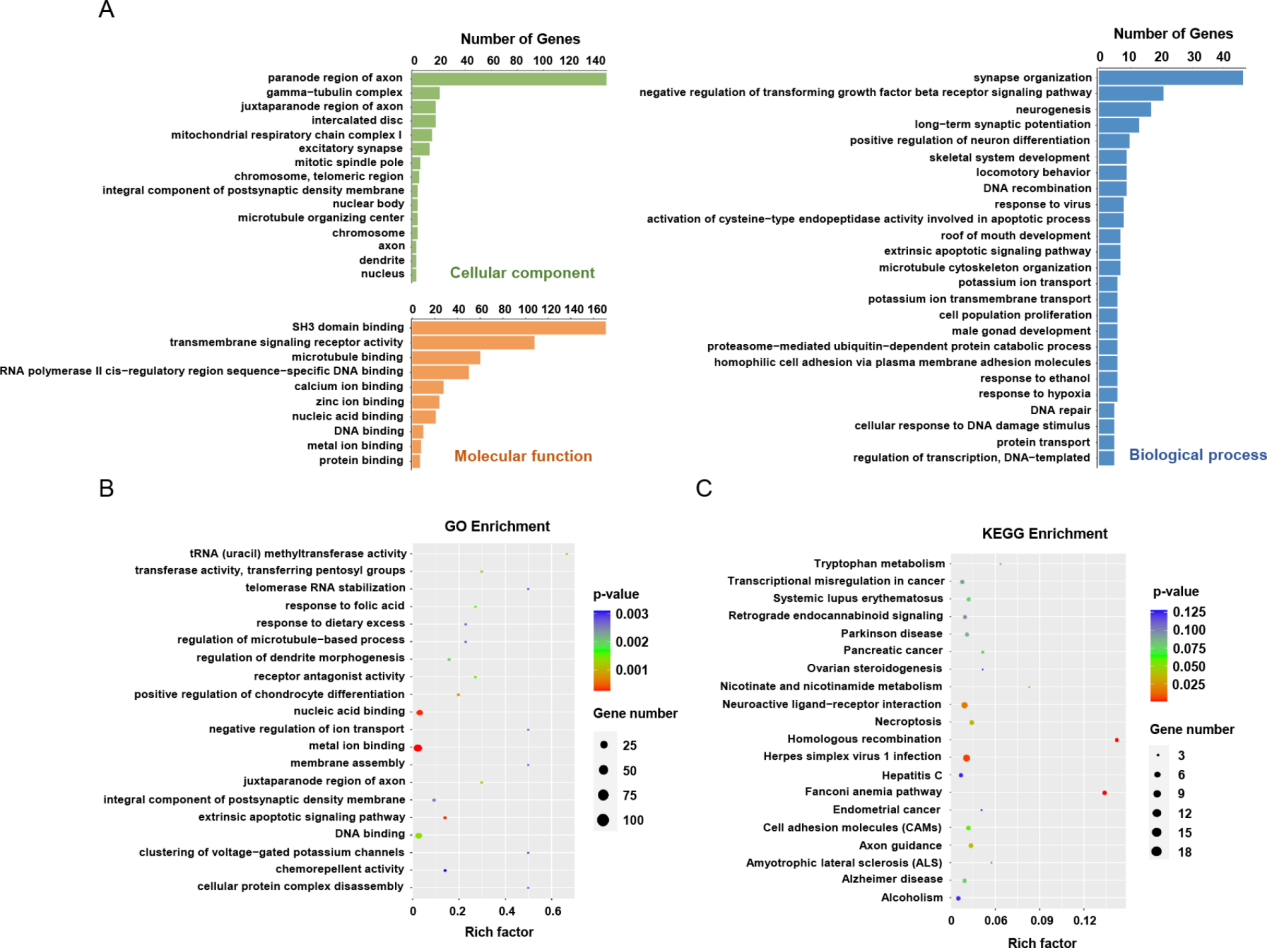
GO function and KEGG pathway enrichment of differentially methylated m^6^A genes. **(A)** Major enrichment and meaningful GO terms of differentially methylated m^6^A genes in AS group relative to that of control. **(B)** The top twenty significant GO enrichment terms. **(C)** The top twenty significant KEGG pathways

To explore the biological significance of m^6^A modification in AS, GO and KEGG pathway analysis were conducted on differentially methylated m^6^A genes. Through GO analysis, three functional domains were divided: cellular component, molecular function, and biological process. Figure 4A lists the top 15 cellular component terms, top 10 molecular function terms, and top 25 biological processes of differentially methylated m^6^A genes. Further analysis showed that those genes were significantly enriched in metal ion binding, nucleic acid binding, and DNA binding (Fig. 4B). The results of KEGG pathway analysis showed that the differentially methylated m^6^A genes were primarily involved in the Fanconi anemia pathway and Herpes simplex virus 1 infection (Fig. 4C). Taken together, these functional prediction analyses might provide primary evidence for investigating the role of a specific m^6^A-modified RNA in AS.

### Integrated analysis of MeRIP-seq and RNA-seq data

**Fig. 5:**
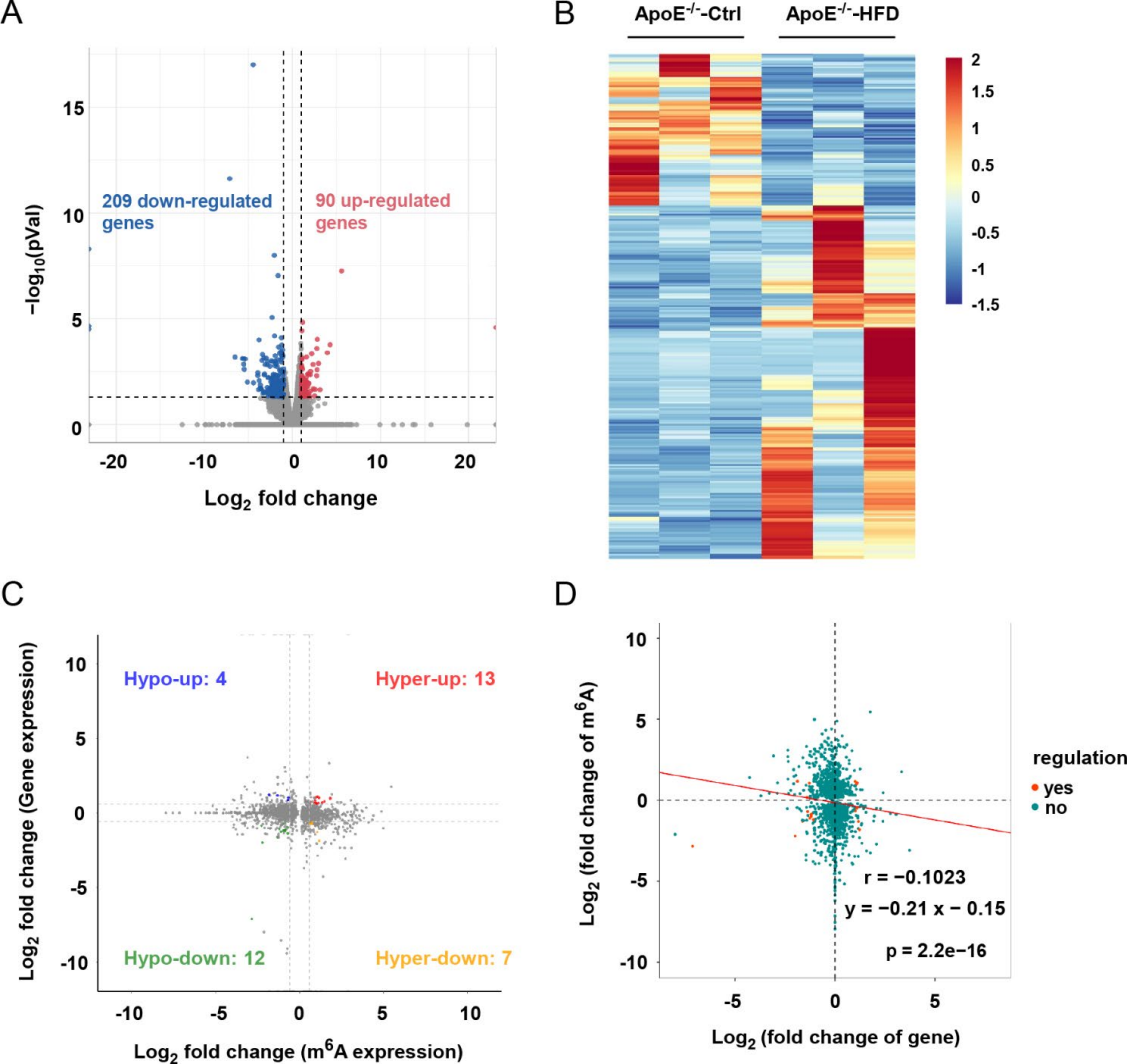
Comprehensive analysis of MeRIP-seq and RNA-seq data. Scatter plot **(A)** and Hierarchical clustering **(B)** showing the differentially expressed genes in AS group. **(C)** Four quadrant graph showing the distribution of genes with a significant change in both m^6^A methylation level and gene expression between AS and control group. **(D)** Negative correlation between overall m^6^A methylation and mRNA expression level (r = -0.1023; y = -0.21x-0.15)

To investigate the differentially expressed genes between AS and control aorta, RNA-seq was simultaneously performed using the MeRIP-seq input library. Our results showed that 299 genes were differentially expressed in AS aorta compare with that of control upon the threshold of |log_2_FC| > 1 and *P* < 0.05 (Fig. 5A, Table S4). Among them, 90 were up-regulated, and 209 were down-regulated. The heat map displayed representative differentially expressed genes between the two groups (Fig. 5B). We then carried out the conjoint analysis of the data from RNA-seq and MeRIP-seq. As shown in Fig. 5C and Table S5, all genes were classified into 4 groups, including 4 hypo-methylated but up-regulated genes (hypo-up), 12 hyper-methylated and up-regulated genes (hyper-up), 13 hypo-methylated and down-regulated genes (hypo-down) and 7 hyper-methylated but down-regulated genes (hyper-down). Our results further showed there was a negative correlation between m^6^A methylation abundance and gene expression level in AS and control samples (Fig. 5D).

Furthermore, GO and KEGG pathway analyses were performed to investigate the functional pathway of differentially expressed genes in AS. It was uncovered that those genes were significantly enriched in AS-relevant biological processes such as phagocytosis recognition, phagocytosis engulfment, and cytokine activity (Figure S1A, Table S7). KEGG pathway analysis showed that differentially expressed genes were significantly enriched cytokine-cytokine receptor interaction and Arachidonic acid metabolism which related to AS (Figure S1B, Table S8). The above results suggest differentially expressed genes may play key roles in AS.

### Confirmation of the partial differentially methylated RNA using MeRIP-qRT-PCR

**Fig. 6:**
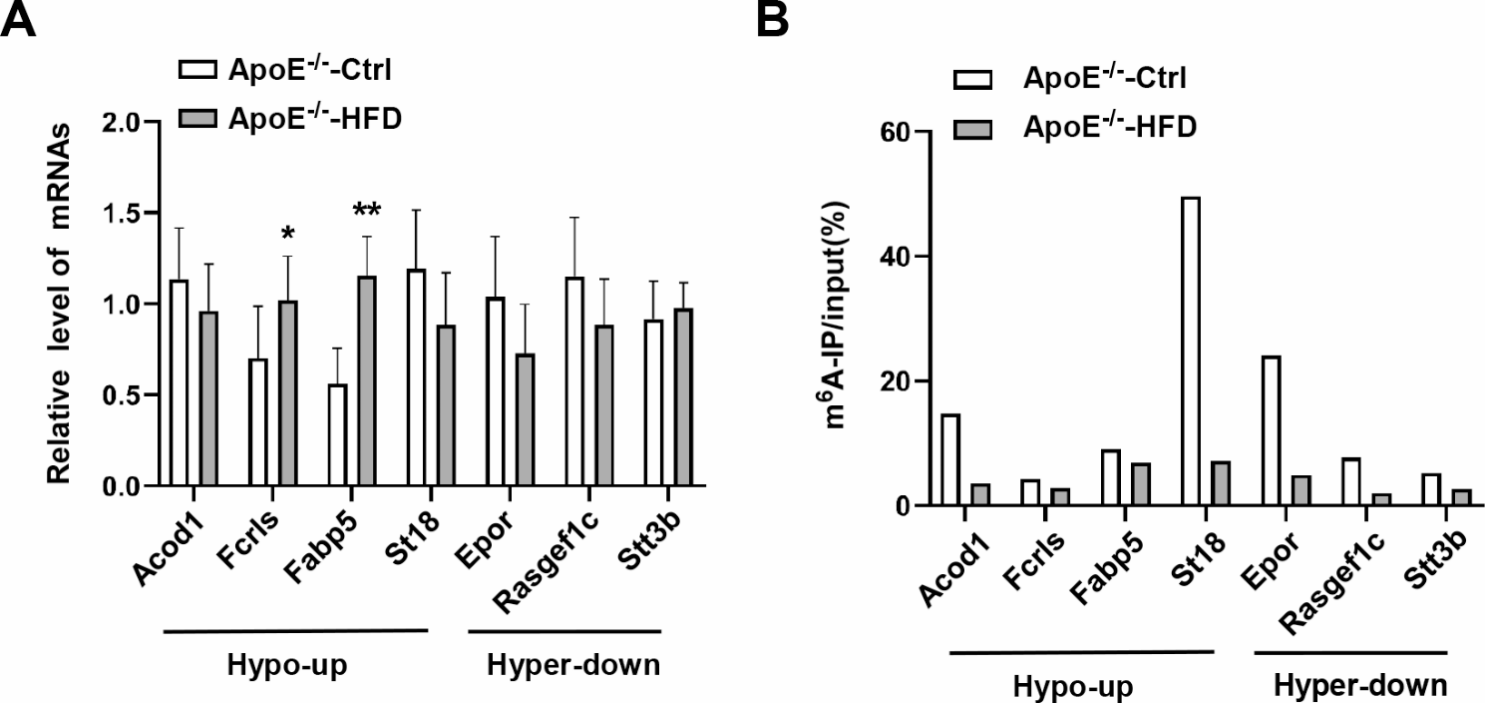
MeRIP-qPCR validation of differentially methylated m^6^A genes. **(A)** The mRNA levels of seven representative differentially methylated m^6^A genes in the aorta were detected by qRT-PCR (n = 7). **(B)** The m^6^A levels of seven representative differentially methylated m^6^A genes in the aorta were analyzed by MeRIP-qPCR (n = 3, three aortic tissues were merged into one sample). All the data are presented as mean ± SD. *P < 0.05, **P < 0.01

To further verify the changes of mRNA m^6^A level obtained from MeRIP-seq, we conducted qRT-PCR analysis in m^6^A-RNA antibody IP (MeRIP) or input samples for some differentially expressed (hypo-up and hyper-down) genes. As shown in Fig. 6A, the mRNA levels of Fclrs and Fabp5 were significantly increased in AS samples which are consistent with the MeRIP-seq data, while the expression of Acod1, St18, Epor, Rasgef1c, and Stt3b did not show a significant difference. MeRIP-qPCR results showed that the m^6^A levels of Acod1, St18, Epor, and Rasgef1c mRNA were significantly decreased (Fig. 6B). These results, in part, confirmed the differential expression and m^6^A modification in AS mice.

## Discussion

As an important post-transcriptional modification, m^6^A modification has attracted increasing attention in recent years. Studies have identified that abnormal m^6^A modification is functional in cardiovascular diseases such as cardiac hypertrophy, myocardial infarction, and heart failure. Although m^6^A-related enzymes had been demonstrated to be involved in regulating the development and progression of AS, the transcriptome-wide distributions of m^6^A modification of AS and its role in AS remained largely unknown. In the present study, we first elucidate the distribution of m^6^A modification in AS mice.

First, in the present study, we observed the m^6^A levels in aorta of AS mice were significantly increased, which was consistent with the increased m^6^A levels in PBMCs from patients with coronary heart disease [15]. Due to the increased m^6^A levels in aorta of AS mice and m^6^A modification is executed by the core m^6^A writer (METTL3, METTL14, and WTAP), we then detect the expression of METTL3, METTL14, and WTAP in aorta. Our results showed that the expression of METTL3 was significantly increased in the aorta of AS mice, which is consistent with a previous study [18]. Furthermore, METLL3 silencing could hamper atherosclerotic plaque formation by targeting JAK2/STAT3 pathway or Braf mRNA, indicating the critical role of RNA methylation in AS progression [19, 20], although the underlying molecular mechanism, deserves further investigation. Another study has demonstrated that the expression of FTO was significantly decreased in the human carotid artery plaques of early atherosclerosis [1]. Overexpression of FTO could inhibit the formation of atherosclerotic plaques [21]. However, no significant change in FTO was observed in our results, but the expression of ALKBH5 was significantly decreased, which might be due to the difference in AS model. These results, when put together, strongly suggest that m^6^A modification mediated by methyltransferases and demethylases participates in the progression of AS.

Our study is the first to use MeRIP-seq and RNA-seq to analyze m^6^A methylation in the whole transcriptome of aorta in AS mice. Notably, RNA content in the aorta of mice is relatively low (7–31 µg), resulting in less RNA enriched after MeRIP (0.1-3 µg). As thus, we had to combine aortas from three mice as one sample for MeRIP-qPCR experiments (as shown in Fig. 6B). The less RNA sample brings difficulty to analyze the transcriptome m^6^A methylation in AS, as well as to the confirmation assay using MeRIP-qPCR. That is the reason why no study used AS sample to conduct epitranscriptomics study until now.

In addition, our m^6^A methylation profile in AS provides specific m^6^A modification sites of differentially expressed genes, laying down a foundation for subsequent studies on the role of these m^6^A-modified genes in regulating their expression and the progression of AS. Many signaling pathways have been demonstrated to be involved in modulating the progress of AS [22–24]. In our study, we identified many critical biological pathways related to differentially methylated genes through GO, such as metal ion binding, nucleic acid binding, and DNA binding. These biological functions are closely related to the onset and development of AS. For instance, many RNA-binding proteins (RBPs) are abnormally expressed in carotid plaques, these RBPs can control different cellular phenotype changes that may determine not only the initiation of atherosclerotic plaque but also the progression toward plaque rupture [25–27]. Ning et al. demonstrated that the DNA-binding protein TDP43 can exacerbate atherosclerosis progression by promoting inflammation and lipid uptake of macrophages [28]. Furthermore, our KEGG analysis showed that genes with differential m^6^A modification were significantly enriched in the Fanconi anemia pathway and Herpes simplex virus 1 infection, which was related to AS. A previous study has revealed that the Fanconi anemia pathway might regulate the proliferation and apoptosis of coronary endothelial cells and smooth muscle cells that are associated with atherosclerotic pathology [29]. Furthermore, a meta-analysis reported that herpes simplex virus type 1 (HSV-1) and type 2 (HSV-2) infection increases AS risk [30]. Therefore, it can be assumed that modulating the m^6^A modification of the transcripts of genes in the Fanconi anemia pathway and Herpes simplex virus 1 infection might provide a new target for AS treatment.

As the most common epigenetic modification in RNA, m^6^A has been shown to function as an important mediator in AS [4, 31, 32]. A recent study has demonstrated that FOXO1 m^6^A methylation could promote its mRNA expression and the development of endothelial cell inflammation and atherosclerosis [14]. Additionally, the methylation of NPC1L serves to facilitate the progression of atherosclerosis [33]. In our study, we identified a significant hypo-methylated gene Fabp5. Fatty acid-binding proteins (FABPs) were originally described as intracellular proteins that can affect lipid fluxes, metabolism, and signaling within cells [34]. As a member of the Fabp family, Fabp5 plays a key role in lipid-related metabolic processes, cell signaling, cell growth, and differentiation, as well as regulating inflammation [35–37]. A previous study has shown that serum Fabp5 concentration is a potential biomarker for residual risk of atherosclerosis [37]. Further study has revealed that Fabp5 has been demonstrated as a sensitive marker of lipid-rich macrophages in the lumen side of atherosclerotic lesions [38]. Our MeRIP-seq results showed that the m^6^A level of Fabp5 mRNA in the aorta of AS mice was significantly decreased accompanied by an increase in Fabp5 mRNA expression. Therefore, it may suggest that Fabp5 may affect AS progression through an m^6^A-dependent regulatory manner.

In addition to coding genes, our study also found that many long noncoding RNAs (lncRNAs) in AS mice also had m^6^A modifications. Among them, we focus on lncRNA-NEAT1, which has been shown to play an important role in AS. Previous studies have found that knockdown of lncRNA-NEAT1 can hinder the development of AS by inhibiting ox-LDL-induced inflammatory response, lipid uptake, and oxidative stress in macrophages [39–41]. Furthermore, lncRNA-NEAT1 can also participate in the regulation of AS progression by regulating ox-LDL-induced endothelial cell injury [42, 43]. Recently, Yang et al. demonstrated that exercise can down-regulate NEAT1 expression through m^6^A modification, thereby inhibiting ox-LDL-induced endothelial cell pyroptosis and AS progression [44]. This study also found that NEAT1 m^6^A modification was significantly increased in endothelial cells after ox-DLD treatment, and further verified the specific site of m^6^A modification. In our study, we also found that the m^6^A modification site of NEAT1 was 5,845,386–5,845,476 in AS mice by MeRIP-seq, and the m^6^A level of NEAT1 was also significantly increased but the |log_2_FC| did not reach > 1 (log_2_FC = 0.29, *P* < 0.05). The regulation of NEAT1 m^6^A modification and expression in AS indicates its functional study is deserved in future.

One limitation of the present study is the relatively low RNA content in mice aortas after MeRIP results in insufficient confirmation of differential expression and modification. Hence, using a more efficient RNA extraction kit (such as PureLink RNA Mini Kit) or using multiple aortas to merge into one sample is helpful to improve the RNA content after MeRIP and the accuracy of experimental results.

## Conclusion

In summary, we profiled transcriptome-wide m^6^A methylome in AS relative to the corresponding normal control and demonstrated a strong association between m^6^A modification and AS progression. Based on our current results screening candidate genes for further functional and mechanism studies is necessary. Our study lays a foundation for further exploring the pathogenesis of AS and provides a new direction for the treatment of AS.

## Materials and methods

### Animal

Healthy male C57BL/6 and ApoE^−/−^ mice (6 weeks old) were purchased from Caven’s experimental animal company (Changzhou, China). Mice were kept at 25 ℃, under a 12 h light-dark cycle, food and water were freely accessible. All animal experimental procedures were approved by the Institutional Animal Care and Use Committee of Hainan Provincial Hospital of Traditional Chinese Medicine (grants number: IACUC-HPHCM-2,203,011).

After genotype identification, we began to establish a model of atherosclerosis in mice fed with high-fat diet for 26 weeks. Then we sacrificed the mice with over-dose anesthetic and collected the whole aorta for further studies.

### m^6^A quantification

The global m^6^A levels were detected by EpiQuik™ m^6^A RNA Methylation Quantification Kit (EpiGentek, NY, USA) according to the manufacturer’s instructions. The absorbance value of each well was measured by a microplate reader (Tecan, Switzerland) at 450 nm, and then calculations were performed based on the standard curve to quantify the m^6^A levels.

### MeRIP-seq and RNA-seq

The MeRIP-seq was performed by LC-Bio Technology Company (Hangzhou, China). Briefly, the total RNA of all aorta samples was isolated and purified using Trizol reagent (Invitrogen, Carlsbad, CA, USA) in accordance with the manufacturer’s procedures. Fifteen micrograms of total RNA in each sample were used, and ribosomal RNA was depleted using Epicentre Ribo-Zero Gold Kit (Illumina, San Diego, CA, USA) according to the product manual. After purification, the ribosomal-depleted RNA was fragmented into small pieces using Magnesium RNA Fragmentation Module (NEB, cat.e6150, USA) under 86 ℃ for 7 min. Half of the RNA fragment was used to construct a strand-specific RNA library in accordance with a dUTP method. The rest fragmented RNA was incubated with m^6^A-specific antibody (No. 202,003, Synaptic Systems, Germany) in immunoprecipitation (IP) buffer for 2 h at 4 ℃. Eluted m^6^A -containing fragments (IP) and untreated input control fragments were converted to final cDNA library following a strand-specific library preparation by dUTP method. The average insert size for the final cDNA library was 300 ± 50 bp. At last, we performed the 2 × 150 bp paired-end sequencing (PE150) on an Illumina Novaseq™ 6000 at LC-BIO Bio-Tech.

### Data analysis of MeRIP-seq and RNA-seq

HISAT2 (http://daehwankimlab.github.io/hisat2) was used to map reads to the reference genome musculus (Version: v101). Mapped reads of IP and input libraries were provided for R package exomePeak, which identifies m^6^A peaks with bed or bigwig format that can be adapted for visualization on the IGV software (http://www.igv.org). MEME and HOMER were used for de novo and known motif finding followed by localization of the motif with respect to peak summit. Called peaks were annotated by intersection with gene architecture using R package ChIPseeker. Then StringTie was used to perform expression level for all mRNAs from input libraries by calculating FPKM (total exon fragments /mapped reads (millions) × exon length (kB)). The differentially expressed lncRNAs and mRNAs were selected according to the criteria of |log_2_ fold change (FC)| >1 and *P* value < 0.05.

### Bioinformatic analysis of differentially expressed and methylated m^6^A genes

Gene Oncology (GO) functional enrichment (http://www.geneontology.org/) and Kyoto Encyclopedia of Genes and Genomes (KEGG) (http://www.kegg.jp/) pathway analysis [45, 46] were conducted on genes with differentially expressed and m^6^A-modified peaks. Regarding functional enrichment, GO is divided into three functional domains: biological process (BP), cellular component (CC), and molecular function (MF). The SRMAP database (http://www.cuilab.cn/sramp) was applied to predict the m^6^A modification site in genes. All the functional terms were regarded as significant as the threshold of *P*-value < 0.05.

### Western blot analysis

RIPA lysate (Beyotime, Shanghai, China) was used to obtain total proteins from the mice aorta. Then a 30 µg protein sample was loaded in 12% SDS-PAGE gel and transferred onto 0.22 μm polyvinylidene difluoride membranes. The membrane was blocked with 5% milk at RT, and then incubated with antibodies against β-actin (1:5000, Proteintech, 20536-1-AP), METTL3 (1:1000, abclonal, A19079), METTL14 (1:1000, abclonal, A8530), WTAP (1:1000, Cell Signaling Technology, 56,501 S), FTO (1:1000, abclonal, A3861), and ALKBH5 (1:1000, abclonal, A11684) overnight at 4℃, β-actin antibody was used as control. Membranes were visualized with an enhanced chemiluminescent detection kit (NCM Biotech, Suzhou, China), and densitometric analysis was performed by image software (Tanon, Shanghai, China). All experiments were repeated at least three times.

### RNA extraction and qRT-PCR analysis

Total RNA was extracted from the mice aorta using Trizol reagent according to the manufacturer’s instructions. In short, 1000 ng of total RNA was reverse transcribed to cDNA using FastKing gDNA Dispelling RT SuperMix (Tiangen, Beijing, China). The cDNA was subjected to real-time PCR using SYBR Green (Bio-Rad, California, USA) according to the manufacturer’s instructions. GAPDH was used as a normalized control. The fold changes of genes were calculated by using the 2^−ΔΔCt^ analytic method. The sequences of primers were listed in Table S1. All experiments were repeated at least three times.

### MeRIP-qRT-PCR

The riboMeRIP m^6^A transcriptome profiling kit (RiboBio, Guangzhou, China) was adopted for the MeRIP-qRT-PCR analysis according to the manufacturer’s instructions. In brief, total RNA was extracted from the aorta and incubated with m^6^A or IgG antibody-conjugated magnetic beads after RNA fragmentation. IgG was used as a negative control. After the incubation, the magnetic beads were placed in the elution buffer to collect RNA. Finally, the m^6^A-bound fraction RNA was recovered using HiPure serum/plasma miRNA kit (Magen, Guangzhou, China), and the m^6^A enrichment of mRNA was determined by qRT-PCR.

### Statistical analysis

Data were represented as means ± SD. Student’s t test (two-tailed) was applied to analyze the data between two groups, and one-way analysis of variance (ANOVA) followed by Tukey’s post hoc test was adopted for analysis among multiple groups. *P* value < 0.05 was significant statistically. SPSS 23.0 were utilized for statistical analysis.

### Electronic supplementary material

Below is the link to the electronic supplementary material.

Supplementary Material 1

Supplementary Material 2

Supplementary Material 3

Supplementary Material 4

Supplementary Material 5

Supplementary Material 6

Supplementary Material 7

Supplementary Material 8

Supplementary Material 9

Supplementary Material 10

## Data Availability

MeRIP-seq and RNA-seq data have been deposit in NCBI SRA database (Accession number: PRJNA990838). To access the data go to page https://www.ncbi.nlm.nih.gov/sra/?term=PRJNA990838, data will be available as soon as the article is published online. The original contributions presented in the study are included in the article/Supplementary Material, further inquiries can be directed to the corresponding author/s.
