# Supplementary figures and images for: Transcriptome-wide N6-methyladenosine methylation profile of atherosclerosis in mice
Source: BMC Genomics. 2023 Dec 14;24:774. doi: 10.1186/s12864-023-09878-1 (PMC10720251; doi:10.1186/s12864-023-09878-1)

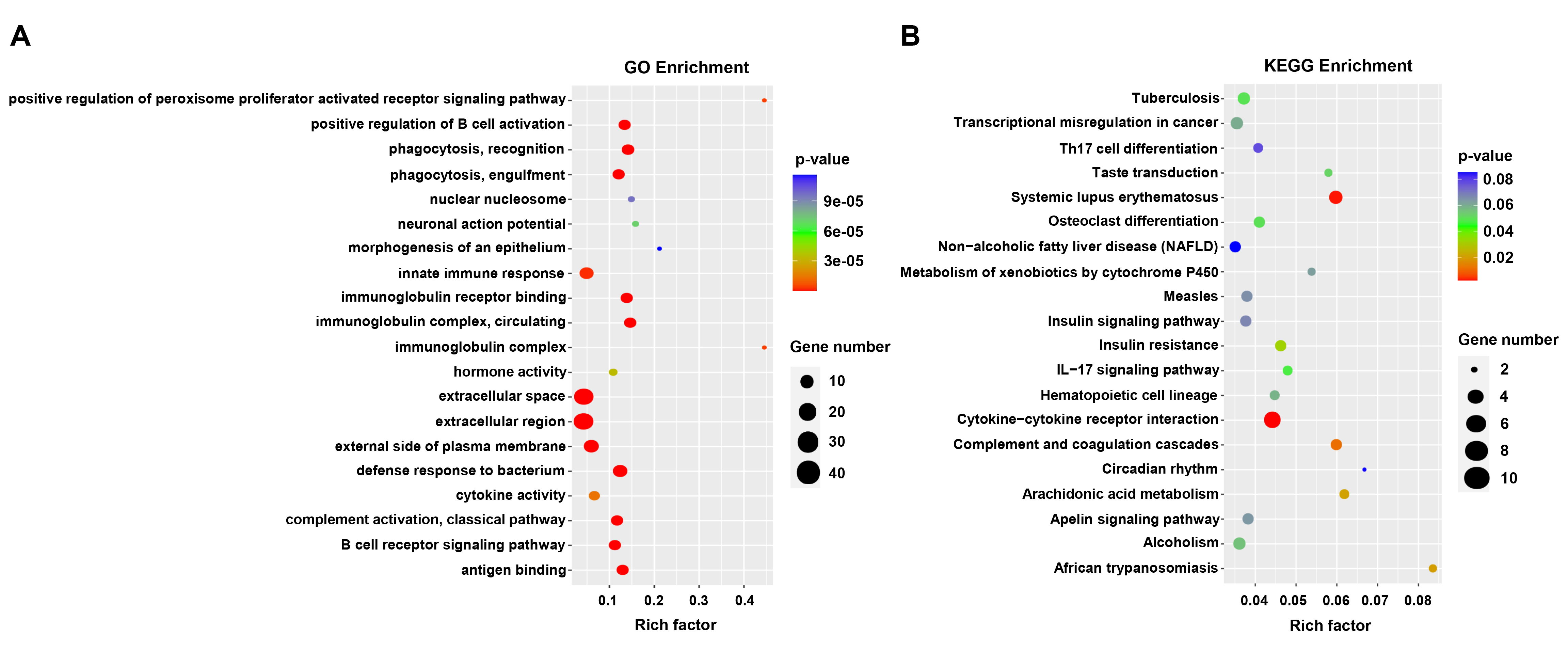

Supplement: Supplementary file 9 — Supplementary Material 9 [file 12864_2023_9878_MOESM9_ESM.png]

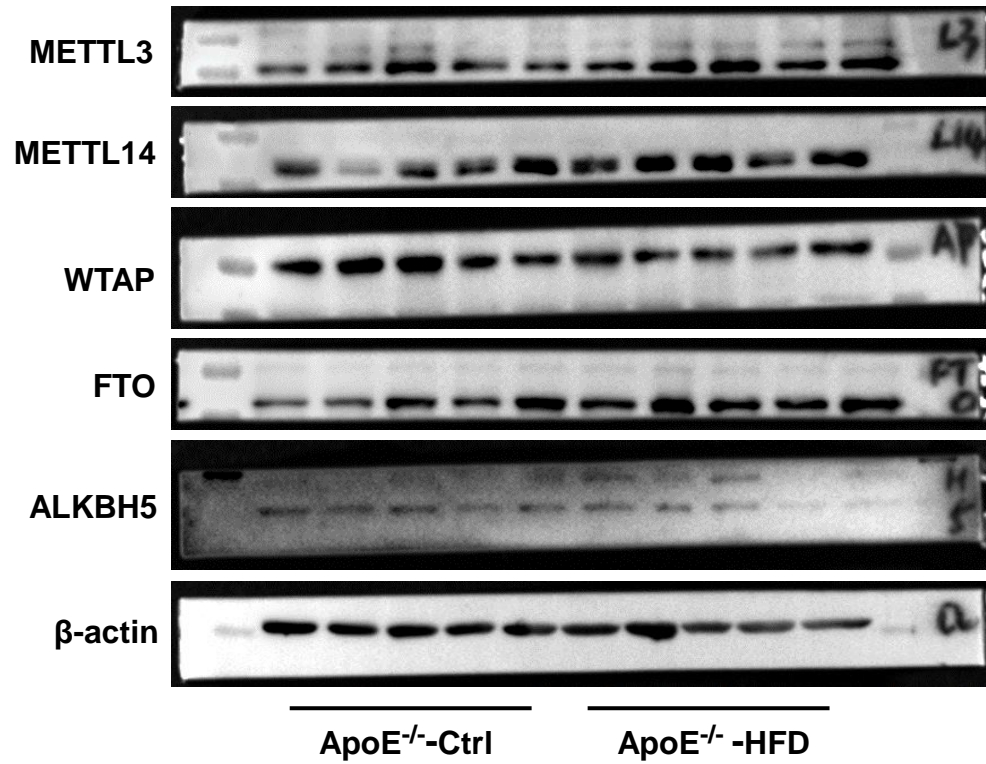

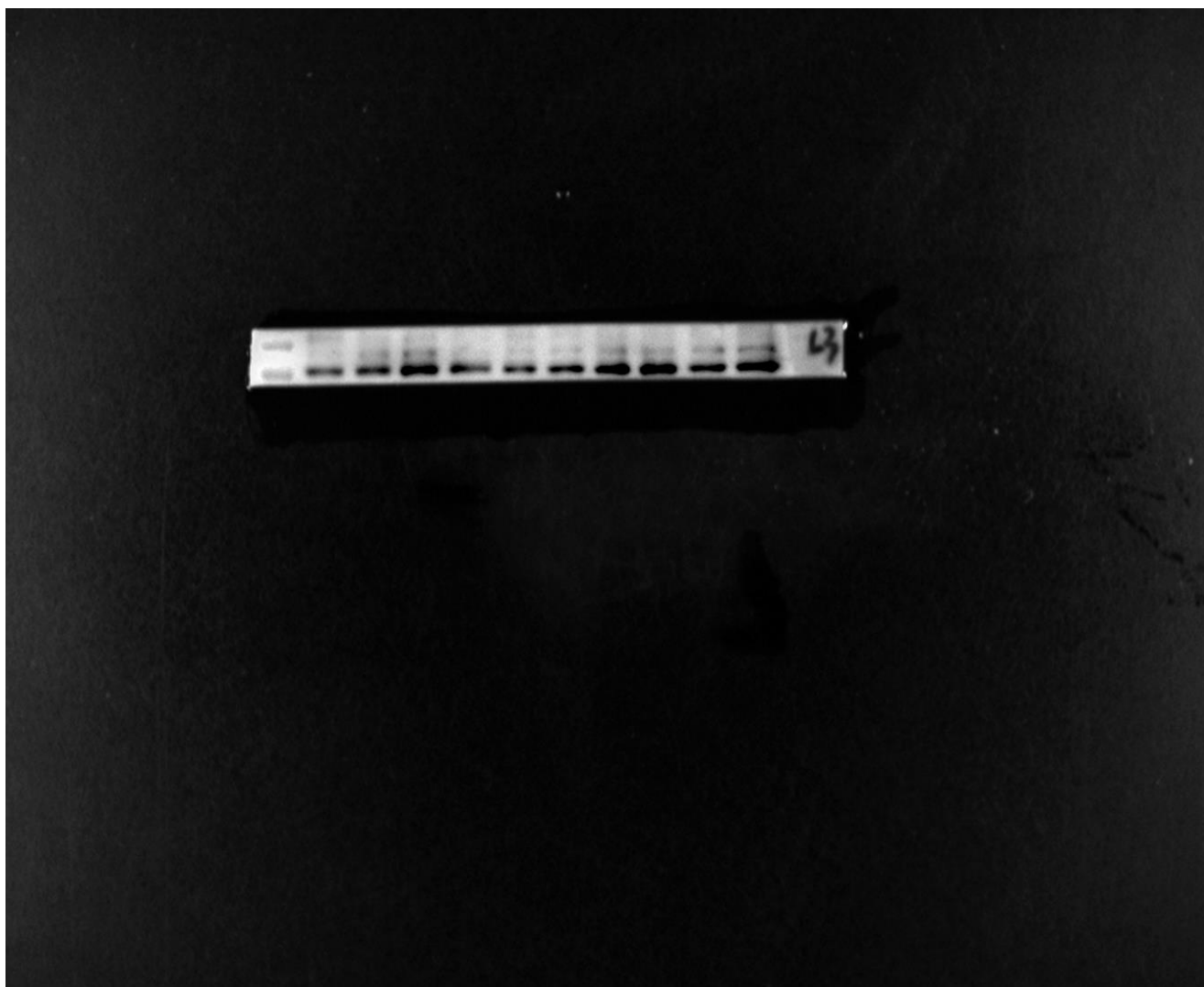

**METTL3**

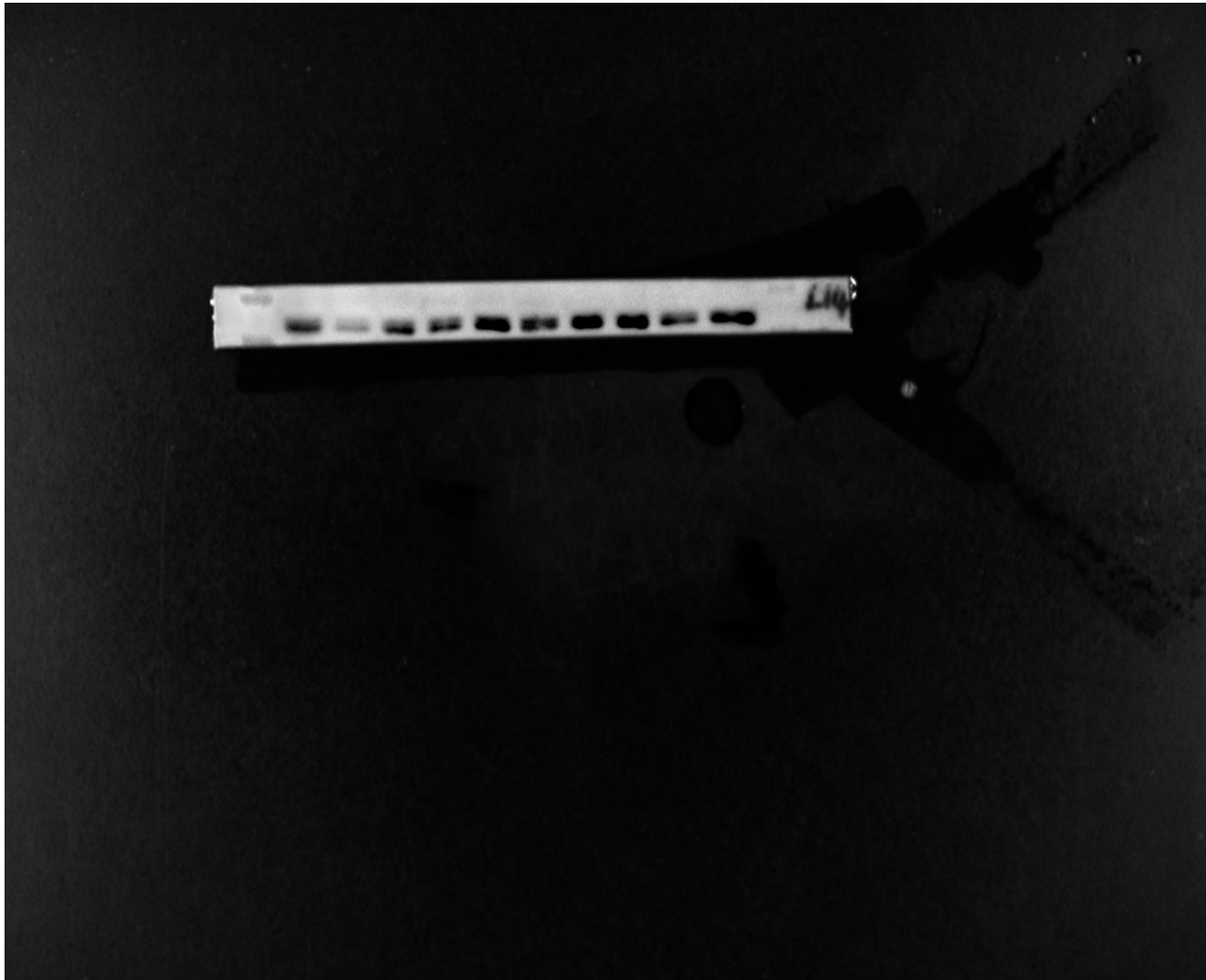

**METTL14**

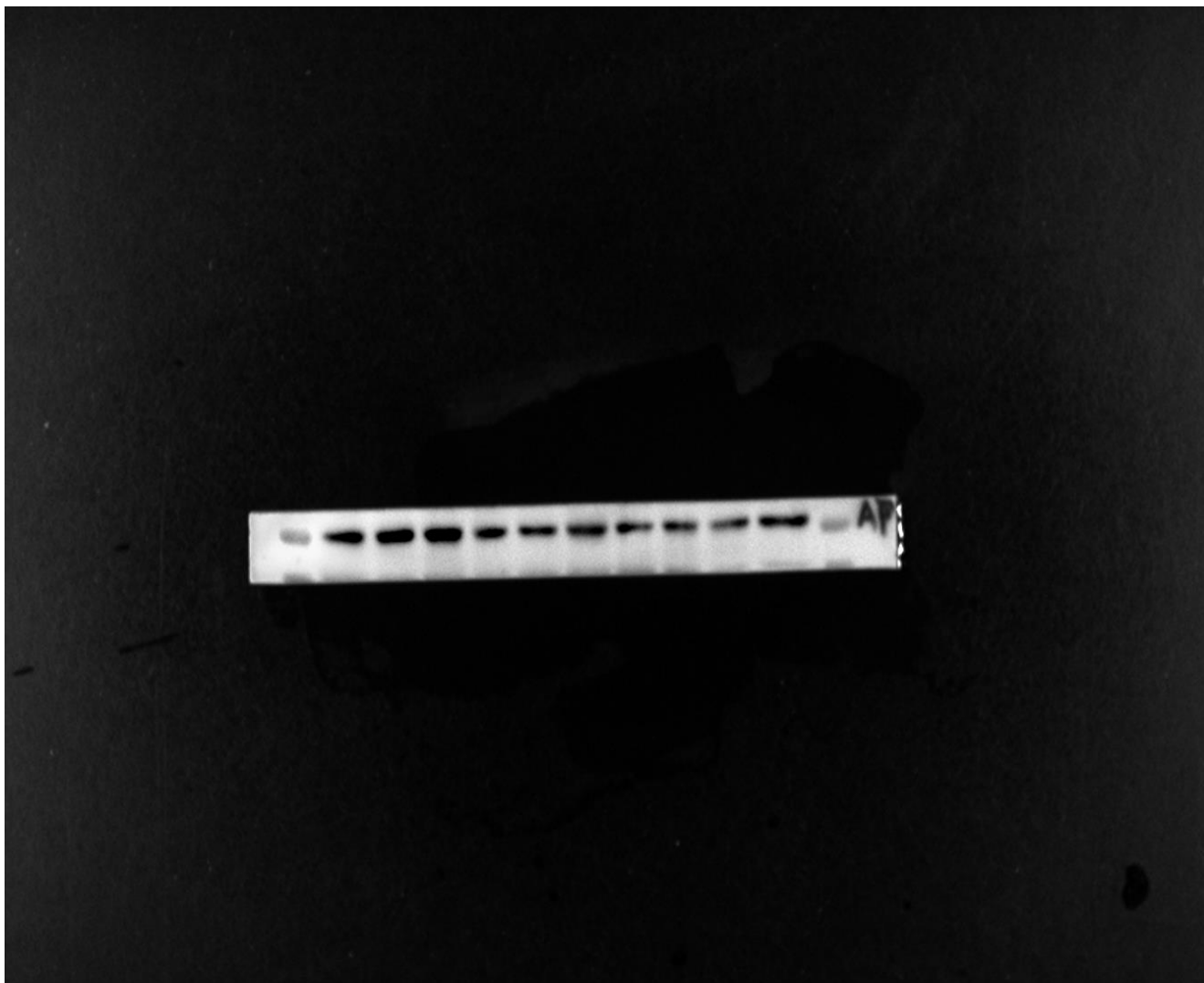

**WTAP**

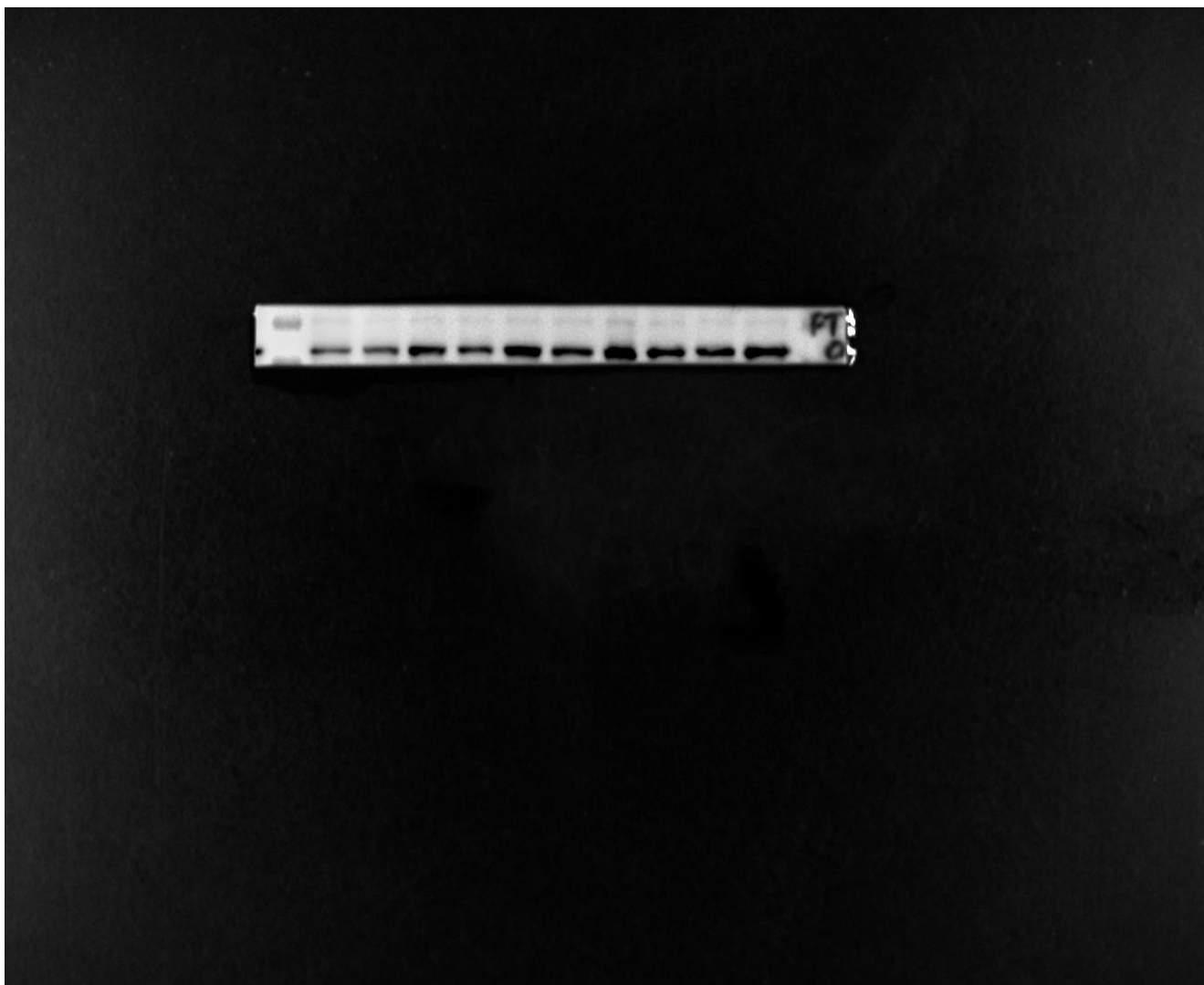

FTO

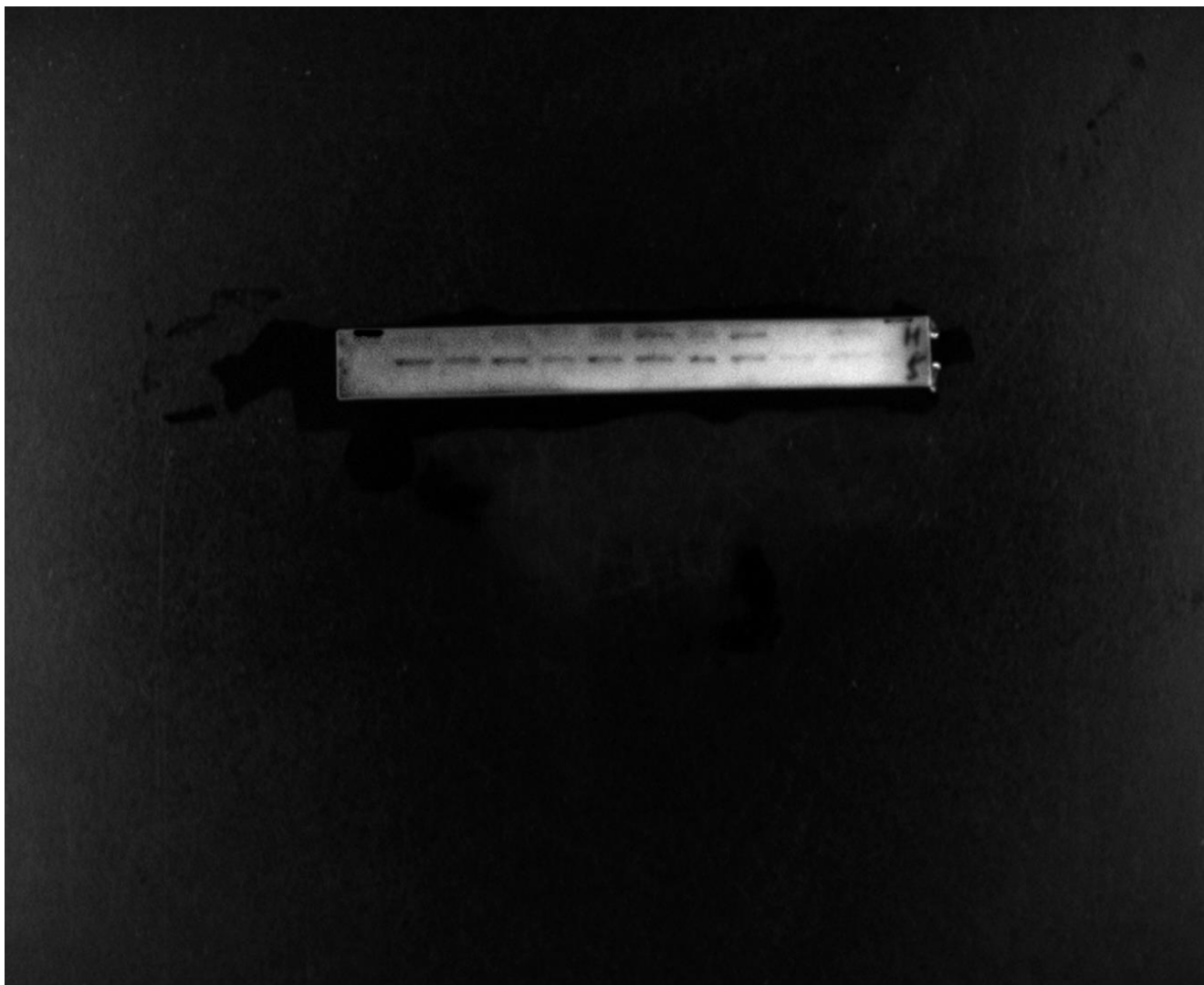

**ALKBH5**

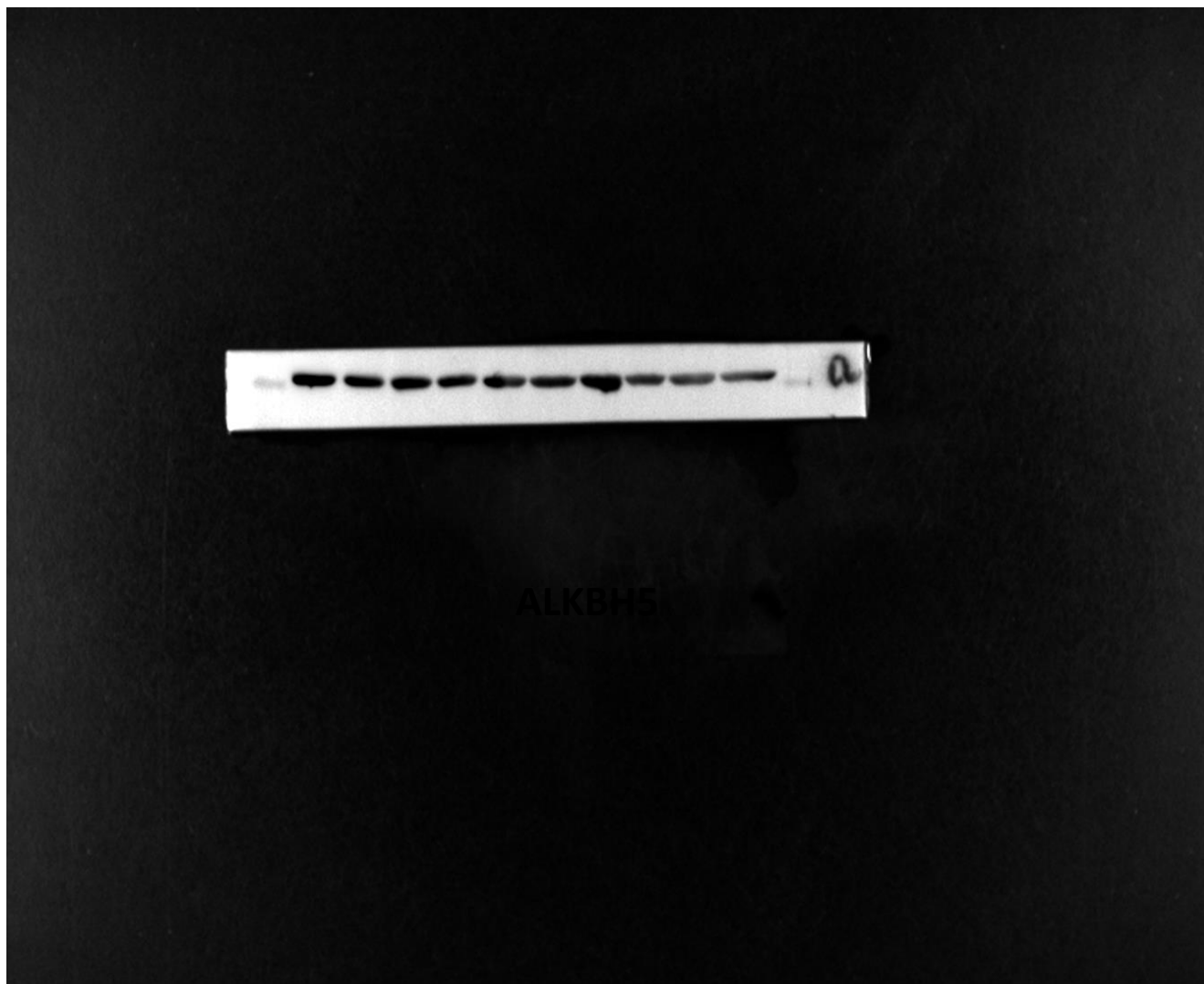

$\beta$ -actin

Supplement: Supplementary file 10 — Supplementary Material 10 [file 12864_2023_9878_MOESM10_ESM.pdf]
